# A method for detecting the rate of tobacco leaf loosening in tobacco leaf sorting scenarios

**DOI:** 10.3389/fpls.2025.1578317

**Published:** 2025-06-05

**Authors:** Yansong Wang, Chunjie Zhang, Mingjie Wu, Ruilin Luo, Lin Lu, Zaiqing Chen, Lijun Yun

**Affiliations:** ^1^ School of Information, Yunnan Normal University, Kunming, China; ^2^ Engineering Research Center of Computer Vision and Intelligent Control Technology, Department of Education of Yunnan Province, Kunming, China; ^3^ Equipment Information Department of Yunnan Tobacco and Leaf Company, Kunming, China

**Keywords:** tobacco leaf loosening rate, image acquisition system, object detection, YOLOv8, detection algorithm

## Abstract

During the tobacco leaf sorting process, manual factors can lead to non-compliant tobacco leaf loosening, resulting in low-quality tobacco leaf sorting such as mixed leaf parts, mixed grades, and contamination with non-tobacco related materials. Given the absence of established methodologies for monitoring and evaluating tobacco leaf sorting quality, this paper proposes a YOLO-TobaccoStem-based detection model for quantifying tobacco leaf loosening rates. Initially, a darkroom image acquisition system was constructed to create a stable monitoring environment. Subsequently, modifications were made to YOLOv8 to improve its multi-scale object detection capabilities. This was achieved by adding layers for detecting smaller objects and integrating a weighted bi-directional feature pyramid structure to reconstruct the feature fusion network. Additionally, a loss function with a monotonic focusing mechanism was introduced to increase the model’s learning capacity for difficult samples, resulting in a YOLO-TobaccoStem model more suitable for detecting tobacco stem objects. Lastly, a tobacco leaf loosening rate detection algorithm was formulated. The results from the YOLO-TobaccoStem were input into this algorithm to determine the compliance of the tobacco leaf loosening rate. The detection method achieved an F1-Score of 0.836 on the test set. Experimental results indicate that the proposed tobacco leaf loosening rate detection method has significant practical application value, enabling effective monitoring and evaluation of tobacco leaf sorting quality, thereby further enhancing the quality of tobacco leaf sorting.

## 1 Introduction

**Figure 1:**
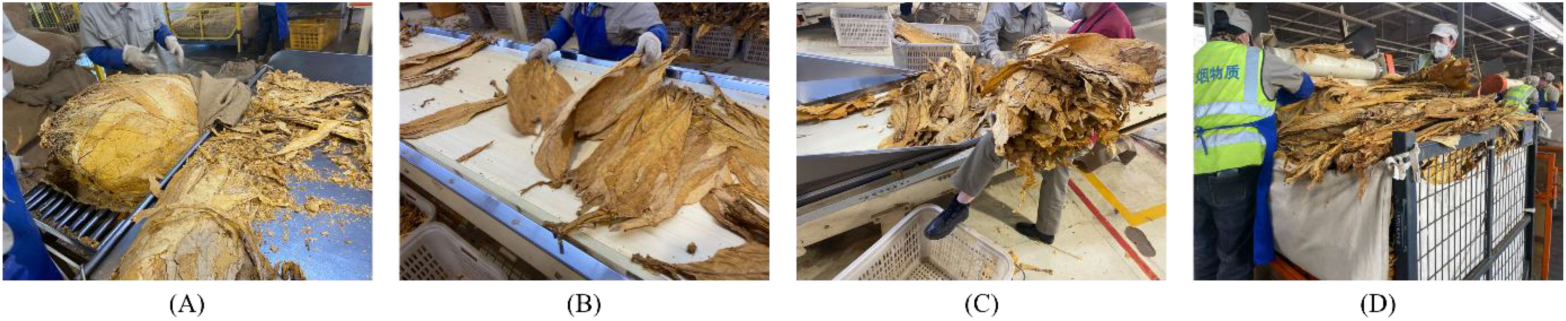
The process of tobacco leaf sorting. **(A)** Tobacco leaf loading: the bundled tobacco leaves are unpacked and placed at the starting position of the conveyor belt by workers. **(B)** Tobacco leaf loosening: workers loosen the overlapping tobacco leaves on the conveyor belt. **(C)** Tobacco leaf grading: workers pick out tobacco leaves of different grades and remove non-tobacco related materials, retaining leaves of the same quality. **(D)** Tobacco leaf packaging: workers pack the sorted tobacco leaves into containers and send them to designated locations for storage.

Tobacco leaves are an important economic crop in China and serve as the primary raw material for cigarette products. As a high-tax and high-profit crop, tobacco leaves hold a significant position in China’s national economy. Thus, it is essential to continuously enhance the economic value of tobacco leaves by improving the quality of cigarette products. The quality of tobacco leaf sorting is a crucial factor affecting the quality of cigarette products. Currently, countries around the world rely primarily on manual methods for tobacco leaf sorting. However, due to differences in workers’ skill levels and varying subjective evaluation standards, the quality of tobacco leaf sorting is often inconsistent. Therefore, it is necessary to strengthen the management of tobacco leaf sorting operations to improve the quality of the sorting process. **Figure 1** shows the process of tobacco leaf sorting.

Tobacco leaf loosening is a crucial step in tobacco leaf sorting. It involves evenly distributing flue-cured tobacco leaves on a conveyor belt, ensuring that the leaves are as separated as possible with no overlap. This process effectively separates foreign matter and different grades of tobacco leaves, thereby improving the purity and quality of the tobacco. Evenly loosened tobacco leaves facilitate subsequent processing and production, ensuring that the final product meets market demands and consumer expectations. If the tobacco leaf loosening is inadequate, it can result in large areas of leaf overlap, leading to issues such as mixed leaf parts, mixed grades, and contamination with non-tobacco related materials. This directly impacts the quality and stability of tobacco leaf sorting, failing to meet the increasingly stringent quality requirements of the cigarette industry. The tobacco leaf loosening rate is an evaluation metric used to assess the effectiveness of the tobacco leaf loosening process. A high loosening rate indicates good performance, meeting the requirements of tobacco leaf sorting, while a low loosening rate indicates poor performance, failing to meet production needs. In summary, as a key step in tobacco leaf sorting, detecting and improving the tobacco leaf loosening rate is vital for enhancing production efficiency and the quality of cigarette products.

In recent years, the development trend of modern agriculture has been shifting towards automation and precision agriculture. Therefore, object detection algorithms that enable automated monitoring and analysis of crops have started to be widely applied in the field of agricultural production. Object detection algorithms are mainly divided into two-stage detection algorithms and one-stage detection algorithms. Two-stage detection algorithms usually include generating candidate regions and performing object classification and localization on these candidate regions. Major examples include R-CNN (Girshick et al., 2014), Fast R-CNN (Girshick, 2015), Faster R-CNN (Ren et al., 2016), and Mask R-CNN (He et al., 2017). While two-stage detection algorithms offer high detection accuracy, they also entail high computational complexity, slow processing speed, and significant computational resource demands, making them unsuitable for scenarios requiring real-time performance or those with limited hardware resources. In contrast, one-stage detection algorithms can directly generate object detection results from the input image, simplifying the detection process and making them more suitable for real-time object detection applications. One-stage detection algorithms primarily include SSD (Liu W. et al., 2016), CENTERNET (Duan et al., 2019), and the You Only Look Once (YOLO) series (Farhadi and Redmon, 2018; Wang A. et al., 2022, 2023). Among these, YOLO algorithms are more widely utilized in agricultural production due to their rapid detection speed and high accuracy, and there is already a body of research in this area. For instance, Jia et al. (2023) developed a new rice pest and disease recognition model based on an improved YOLOv7 algorithm, achieving a detection accuracy of 92.3% on their self-built dataset. Li et al. (2024) proposed a lightweight YOLOv8 network for detecting densely distributed maize leaf diseases, achieving a detection accuracy of 87.5% with a model size of 11.2MB. Zhong et al. (2024) proposed a lightweight mango detection model, Light-YOLO, which achieved better detection results than other YOLO series models while using fewer parameters and FLOPs.

With the rise of Industry 4.0, advanced technologies such as the Internet of Things (Al-Fuqaha et al., 2015), big data (Lv et al., 2014), machine learning (Jordan and Mitchell, 2015), and deep learning (LeCun et al., 2015) have rapidly developed, prompting the tobacco industry to embark on the path of informatization and automation. Traditional tobacco crop production often requires a substantial amount of human resources. However, the introduction of automated detection and control systems can save significant labor costs while reducing various cumbersome issues that arise during production, thereby enhancing the production efficiency of tobacco crops and the quality of cigarette products. Currently, there are some studies in this field. Xu et al. (2023) proposed a multi-channel and multi-scale separable dilated convolution neural network with attention mechanism, achieving an accuracy of 98.4% on their self-built dataset. He et al. (2023) proposed an end-to-end cross-modal enhancement network that extracts multi-modal information to grade tobacco leaves, achieving a final grading accuracy of 80.15%. Huang et al. (2024) proposed a method for single tobacco leaf identification in complex habitats based on the U-Net model. Xiong et al. (2024) developed a deep learning model, DiffuCNN, for detecting tobacco lesions in complex agricultural settings, achieving a detection accuracy of 98%. Tang et al. (2023) studied a method for locating tobacco packaging and detecting foreign objects, proposing a cascade convolutional neural network for detecting foreign objects on the surface of tobacco packages and developed a data generation methodology based on homography transformation and image fusion to generate synthetic images with foreign objects, enhancing their model’s performance, and achieving a final mAP of 96.3%. Wang C. et al. (2022), based on YOLOX and ResNet-18, proposed a real-time production status and foreign object detection method for smoke cabinets, preventing safety and quality issues caused by foreign objects. Liu X. et al. (2016) investigated the hardware selection, parameter setting, and software design of the PLC control system in the tobacco blending control system, improving the quality of cigarettes by adjusting the mixture ratios of various components. Rehman et al. (2019) analyzed the integration and application of machine learning technologies in the field of tobacco production and discussed future trends.

Based on the above content, it is evident that current research primarily focuses on detecting tobacco plants themselves or on the automated grading of tobacco leaves. However, there is a lack of research on the rate of tobacco leaf loosening. Therefore, this paper fully utilizes relevant concepts from the field of computer vision, closely integrating them with the actual conditions of tobacco leaf sorting work, and proposes a method for detecting the rate of tobacco leaf loosening. By detecting the rate of tobacco leaf loosening, real-time supervision of the loosening process can be achieved, allowing for timely adjustments to non-compliant tobacco leaves, thereby improving the quality of tobacco leaf loosening and ultimately enhancing the quality of tobacco leaf sorting. The research mainly faces the following challenges:

1. The tobacco leaf sorting site has uneven lighting and a large amount of dust, causing the captured images to often suffer from issues such as motion blur, making it difficult to obtain standardized images.
2. Loosened unqualified tobacco leaves often exhibit significant overlapping, which can lead to missed detections and duplicate identifications during inspection. The trained model needs to meet industrial deployment standards, which means it must be small in size, fast, and highly accurate.
3. In the field of tobacco research, there is no dataset available for tobacco leaf loosening.
4. The definition of the tobacco leaf loosening rate is unclear, and there is no reasonable method for its calculation.

To address the aforementioned issues, this paper proposes a method for detecting the rate of tobacco leaf loosening. The main contributions of this paper can be summarized as follows:

1. A darkroom image acquisition system was constructed to obtain more stable tobacco leaf images under actual production conditions. To simulate real-world operational environments, a conveyor belt matching factory production line speeds was implemented, with the darkroom image acquisition system installed above it. The fixed-lighting darkroom environment effectively addressed field challenges including uneven lighting conditions and high dust levels. An industrial camera positioned perpendicular to the conveyor belt within the darkroom was deployed. Through preconfigured camera parameter optimization, this configuration enhanced image acquisition stability and ultimately resolved the challenge of acquiring standardized tobacco leaf images.
2. The YOLO-TobaccoStem object detection model was developed to detect tobacco leaves on conveyor belts. Built upon the YOLOv8 framework, the model’s detection capability for smaller objects was enhanced through the integration of a dedicated small-object detection layer. The feature fusion module was reconstructed using a weighted bi-directional feature fusion structure to strengthen multi-scale feature integration. Furthermore, the original loss function was replaced to optimize detection performance for highly overlapping targets. These targeted modifications resulted in the YOLO-TobaccoStem model being better suited for detecting loosened tobacco leaf images in industrial inspection scenarios.
3. The tobacco leaf loosening dataset was established and publicly released to address the absence of specialized data resources in this research domain. Raw tobacco leaf images were acquired using the darkroom image acquisition system, and the dataset was systematically constructed through data preprocessing and augmentation procedures. The creation and publication of this tobacco leaf loosening dataset holds significant implications for tobacco leaf research, as it filled a critical data gap in tobacco leaf loosening rate analysis while providing essential data infrastructure for subsequent studies.
4. A tobacco leaf loosening rate detection algorithm was developed to enable real-time computation and quantitative assessment of tobacco leaf loosening rates. The position information of tobacco stems is obtained from the bounding box coordinates output by the YOLO-TobaccoStem model. Based on this position information, the rate of tobacco leaf loosening is calculated and evaluated, ultimately determining whether the loosening rate of the bunched tobacco leaves meets the required standards.

The remaining of this paper is organized as follows: The “Materials and Methods” section introduces the construction of the dataset and the method for detecting the tobacco leaf loosening rate, which includes the image acquisition system, the YOLO-TobaccoStem object detection model, and the tobacco leaf loosening rate detection algorithm. The “Experiments and Results” section conducts a series of experiments on the proposed model and verifies the effectiveness of the improvements suggested in this paper through experimental results. The “Discussion” section analyzes potential factors that may affect the model, tests the tobacco leaf loosening rate detection algorithm, and discusses the feasibility of the methods. The “Conclusion” section summarizes the study.

## 2 Materials and methods

**Figure 2:**
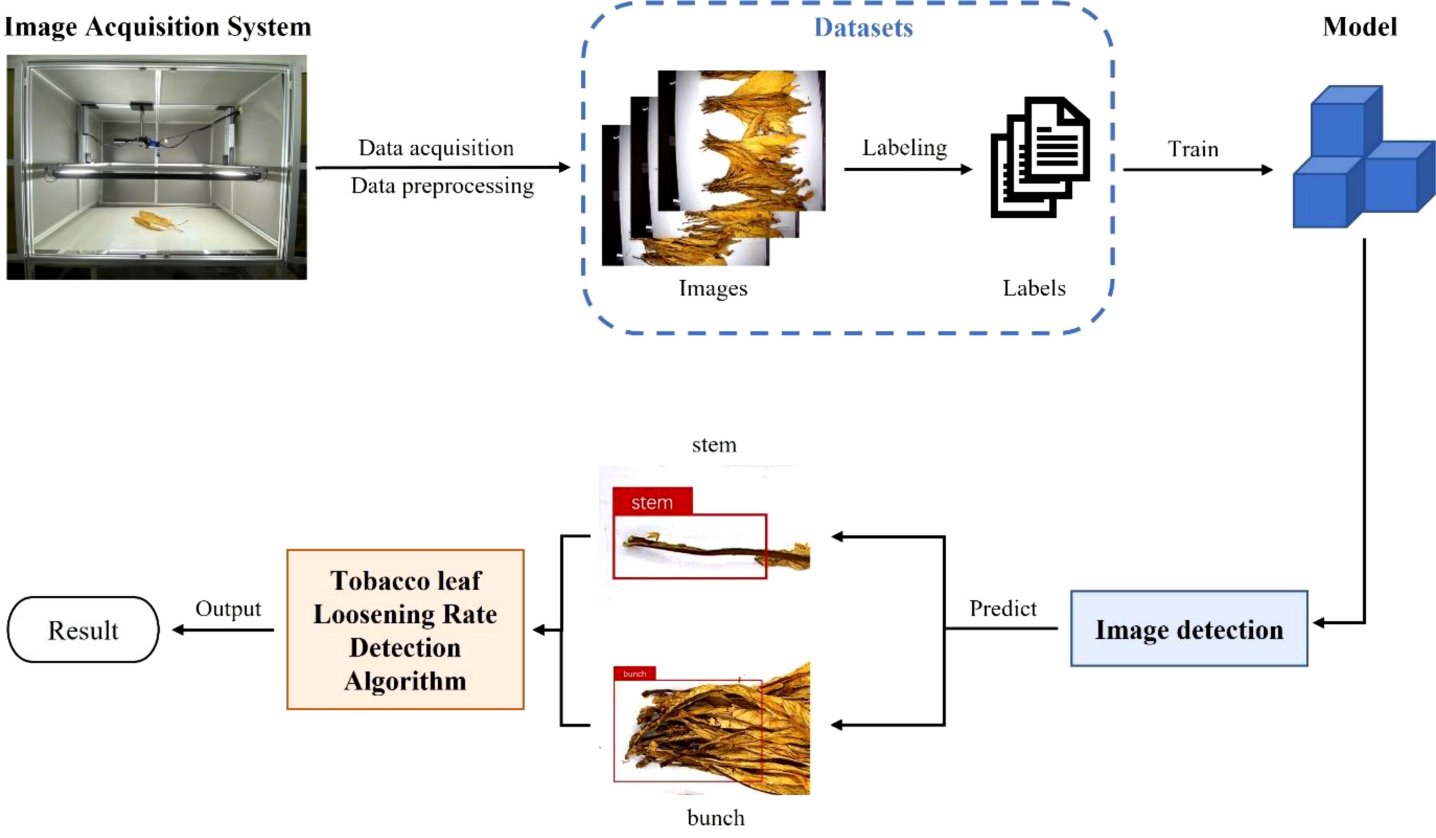
Workflow diagram.

This paper proposes a method for detecting the tobacco leaf loosening rate to monitor and evaluate the quality of tobacco leaf loosening operations. Firstly, to establish a stable image acquisition environment, this study constructed a darkroom image acquisition system. Then, utilizing this image acquisition system, numerous images of loosened tobacco leaves were collected, and a tobacco leaf loosening dataset was created through data preprocessing and data augmentation. Subsequently, to detect loosened tobacco leaves on the conveyor belt, a YOLO-TobaccoStem object detection model specifically optimized for addressing tobacco leaf loosening challenges was developed. Finally, a tobacco leaf loosening rate detection algorithm was proposed to calculate and characterize the loosening rate by processing the detection results from YOLO-TobaccoStem, ultimately determining whether the loosening rate meets quality standards. The workflow of this study is illustrated in **Figure 2**.

### 2.1 Darkroom image acquisition system

To address various challenges encountered in factory environments and establish a stable image acquisition environment, we conducted an in-depth investigation of the tobacco sorting workshop. Based on these findings, the following key factors were prioritized in the design of the darkroom image acquisition system.

#### 2.1.1 Consistent lighting and dust control

The primary objective of the darkroom image acquisition system is to address the challenges posed by uneven lighting and pervasive dust, which hinder the capture of standardized images. Therefore, it is imperative that the design ensures a relatively enclosed environment, equipped with stable and uniform light sources, to facilitate the acquisition of standardized images.

#### 2.1.2 Durability and longevity

An excellent hardware system should have a long lifespan. Considering the temperature, humidity, and other conditions prevalent in the tobacco leaf sorting workshop, the hardware system must resist performance degradation due to environmental changes or prolonged use. Consequently, aluminum alloy, renowned for its low density, high strength, and corrosion resistance, was selected for the external framework of the darkroom image acquisition system.

#### 2.1.3 Ease of operation

Since the system is intended for use in tobacco leaf sorting site, it should be designed for easy operation. This reduces training time for employees, increases production efficiency, minimizes the possibility of operational errors, and ensures the stability of image capture quality.

**Figure 3:**
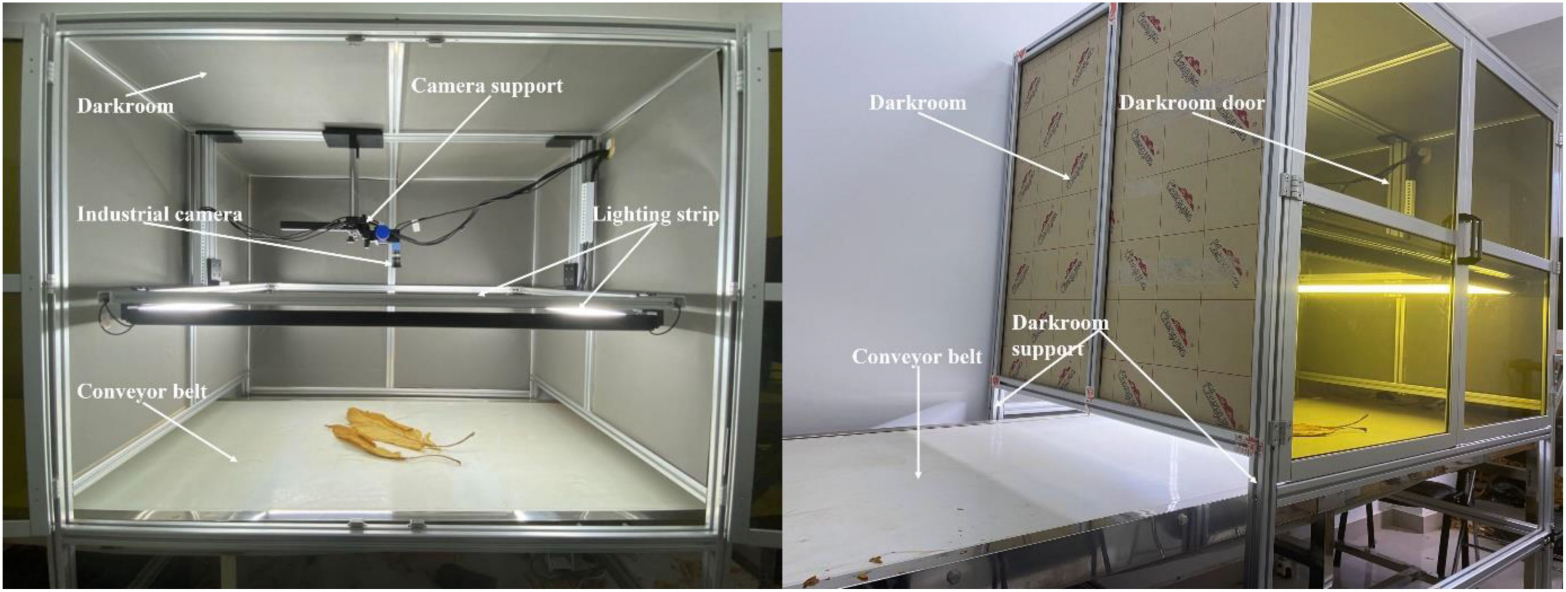
Darkroom image acquisition system.

Based on these requirements, a darkroom image acquisition system, as shown in **Figure 3**, was constructed in a laboratory environment simulating the on-site conditions. The system is mounted on a conveyor belt system that mimics the tobacco leaf sorting site, and it operates normally.

The overall frame of the darkroom is constructed using aluminum alloy supports, which provide structural stability while remaining lightweight for ease of transport and installation. The sides and top of the darkroom are built from light-blocking acrylic panels. Acrylic was selected for its high impact resistance and excellent aging resistance, ensuring long-term durability. Except for the darkroom door, all other acrylic panels completely block external light. The darkroom door is made of tinted acrylic, allowing staff to observe the internal conditions while minimizing the impact of external light on the internal lighting environment.

Inside the darkroom, the primary components are an industrial camera and a lighting system. The lighting system consists of four light strips installed on the sides of the darkroom. These strips are arranged in a rectangular configuration on the supports to ensure even light distribution and maintain internal lighting stability. By adjusting the height of the supports, the light can be kept bright without causing glare on the conveyor belt. The industrial camera is mounted on the top of the darkroom to capture images of the tobacco leaves on the conveyor belt. The MER-503-20GM area scan camera from Daheng Image was chosen for its compact size (29mm × 29mm × 29mm), offering flexibility and convenience for installation and removal. It is robust and can operate in environments ranging from 0°C to 45°C, suitable for the conditions at the tobacco leaf sorting site. The camera’s sensor features a global shutter function, eliminating motion blur during image capture, and it is known for low noise and high stability.

The darkroom image acquisition system designed in this study uses aluminum alloy and acrylic panels as the primary materials. This ensures a long service life for the system while keeping it lightweight and easy to assemble. Once the entire system is set up, it requires minimal adjustments and is ready for immediate use with simple operation. The main function of this system is to effectively mitigate the issues of uneven lighting and pervasive dust in the tobacco leaf sorting site, thereby enabling the capture of standardized tobacco leaf images.

### 2.2 Dataset

#### 2.2.1 Data acquisition

The tobacco leaves used in this study were provided by the Yunnan Tobacco Leaf Company of the China National Tobacco Corporation (Kunming, Yunnan, China). The tobacco leaves were sourced from Dali City, Yunnan Province, and were of grade C3F. The images were collected on 2023. The equipment used for image collection was the darkroom image acquisition system described in Section 2.1.

**Figure 4:**
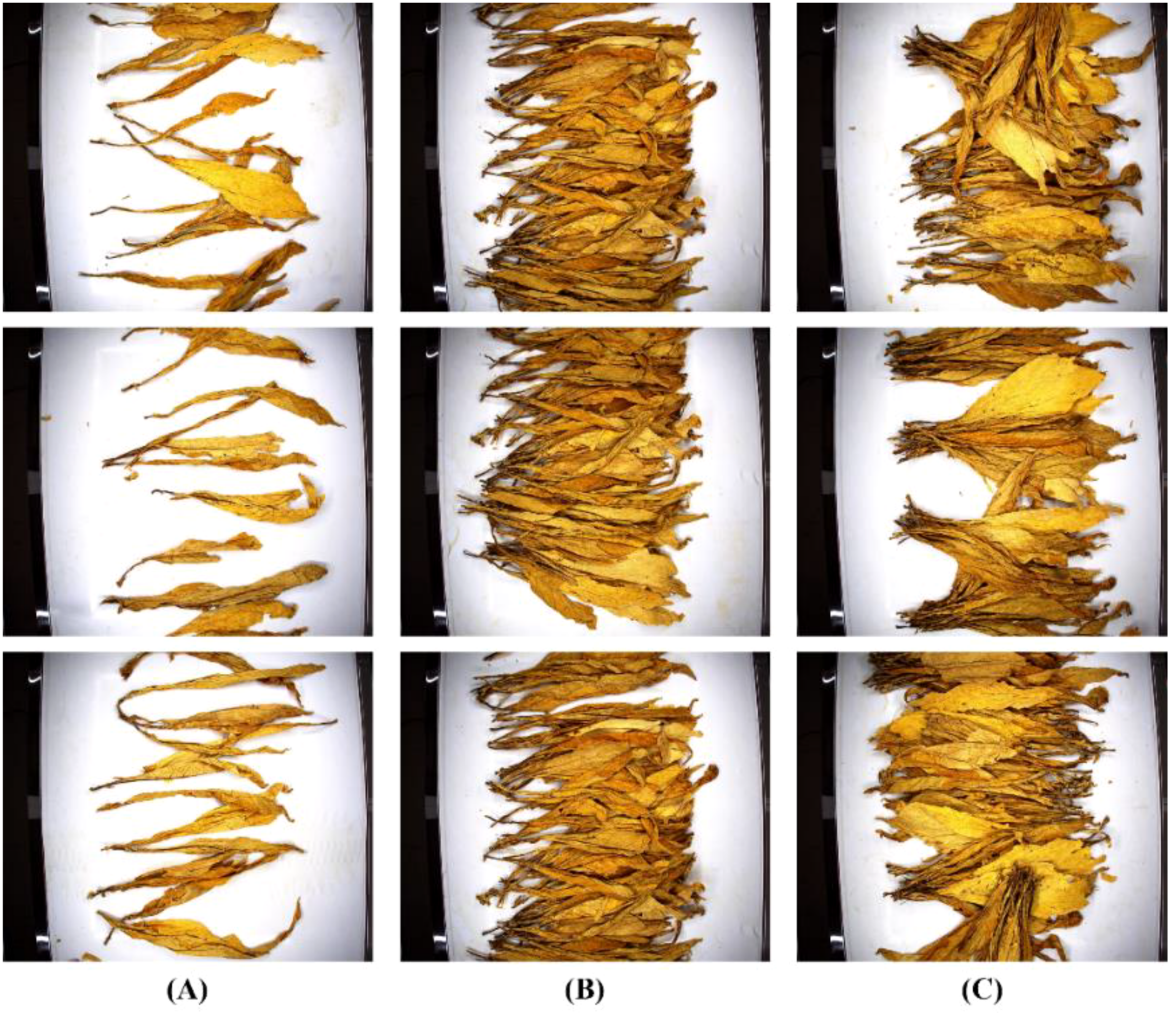
States of loosened tobacco leaves. **(A)** Sparse distribution of tobacco leaves. **(B)** Dense distribution of tobacco leaves. **(C)** Stacked bunches of tobacco leaves.

To simulate a real production environment, a conveyor belt system similar to the production line was set up, and the tobacco leaf loosening process was carried out on the conveyor belt. To ensure the stability of the collected image quality, the conveyor belt speed was set to 0.07 m/s, allowing it to pass through the darkroom at a constant speed. Images of the tobacco leaves were captured using the darkroom image acquisition system, with an output resolution of 2448x2048, saved in JPEG format. **Figure 4** shows different states of tobacco leaf loosening in the dataset.

#### 2.2.2 Data preprocessing

To enhance the detection accuracy and training efficiency of the YOLO-TobaccoStem model, the collected images were first preprocessed to ensure obtaining a high-quality dataset. The collected images of tobacco leaf loosening were manually screened to exclude blurred images and those with too few tobacco leaves, resulting in an initial dataset of 1055 images, encompassing all the states of tobacco leaf loosening mentioned in the previous section. LabelImg (Ke et al., 2024) was used to annotate the tobacco leaf loosening images. Two types of labels were designed: single tobacco stem (stem) and overlapping tobacco stems (bunch), and the labels were saved in txt file format for subsequent training.

To improve the model’s robustness, reduce its sensitivity to images, and enhance its generalization ability, the original dataset underwent data augmentation. The original dataset was first divided into training, validation, and test sets in a 7:2:1 ratio. Then, various data augmentation methods, including rotation, cropping, brightness adjustment, image translation, horizontal flipping, cutout, and adding Gaussian noise, were applied to the training and validation sets. To ensure the accuracy of subsequent experimental results, the test set was not augmented. The final tobacco leaf loosening dataset comprised 2955 images. Detailed information about the tobacco leaf loosening dataset established in this study is illustrated in **Table 1**.

**Table 1:** Detailed information of the tobacco leaf loosening dataset.

Dataset	Number of images	Label name	Number of instances
Train	2217	stem	35934
		bunch	699
Val	633	stem	10449
		bunch	171
Test	105	stem	1726
		bunch	37

**Figure 5:**
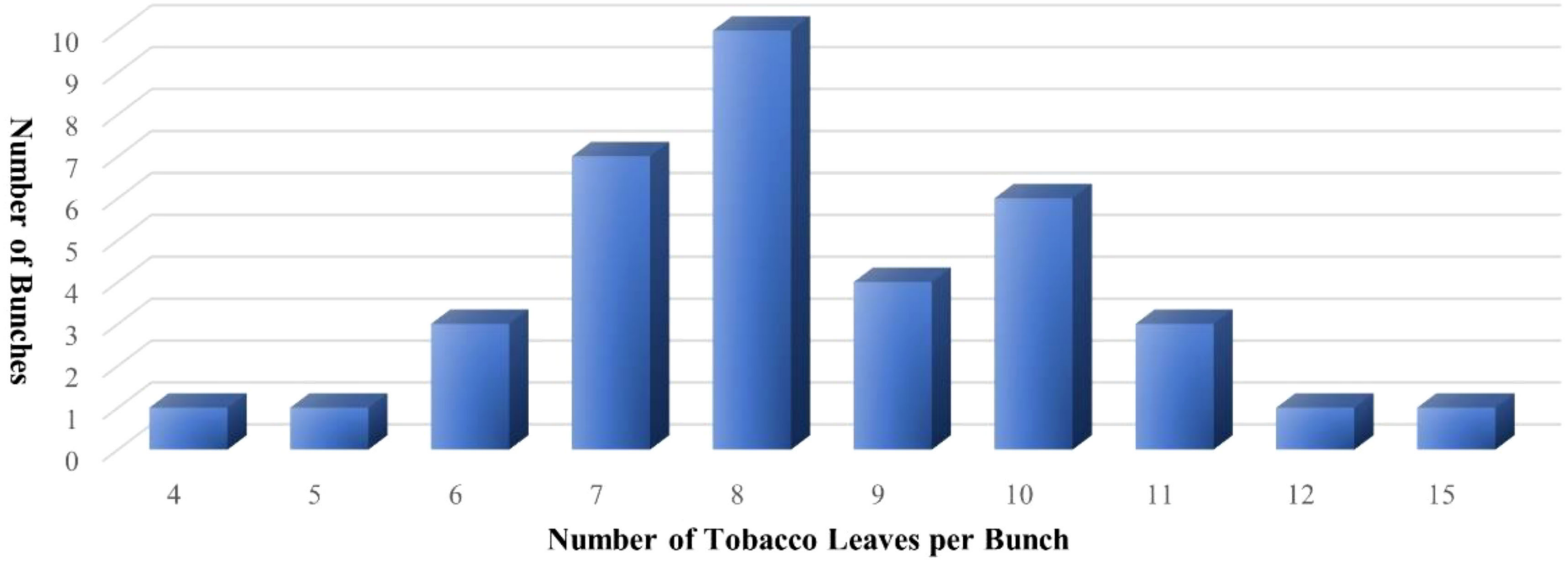
Distribution of the number of tobacco leaves.

The test set includes 37 bunches of tobacco leaves, with the distribution of the number of leaves contained in each bunch illustrated in **Figure 5**.

### 2.3 YOLO-TobaccoStem object detect model

An important part of this study is the design of a lightweight object detection model to provide data support for the subsequent tobacco leaf loosening rate detection algorithm. Based on the YOLOv8 (Jiang et al., 2023) model, we propose the YOLO-TobaccoStem model, which is more suitable for detecting tobacco stems. Compared to other mainstream object detection models, our model achieves the highest detection accuracy while maintaining fewer parameters and a smaller model size. Therefore, this model can effectively provide technical support for the tobacco leaf loosening rate detection method.

#### 2.3.1 YOLOv8

The YOLOv8 object detection algorithm has advantages of higher detection accuracy and better real-time performance compared to other algorithms in the YOLO series. YOLOv8 consists of four main parts: the input end, backbone network, neck network, and detection head. Images enter the network through the input end in a 640×640 base format. The backbone network is responsible for extracting multi-scale feature information from the input images. The neck network then fully integrates the multi-scale feature information extracted by the backbone network using FPN (Lin et al., 2017) - PAN (Liu et al., 2018) methods. Finally, the detection head completes the detection task. YOLOv8 has five model sizes: YOLOv8n, YOLOv8s, YOLOv8m, YOLOv8l, and YOLOv8x, with increasing parameters and model sizes. Considering detection accuracy and real-time performance, we chose the smallest model, YOLOv8n, for our study and made optimizations based on it.

#### 2.3.2 YOLO-TobaccoStem

**Figure 6:**
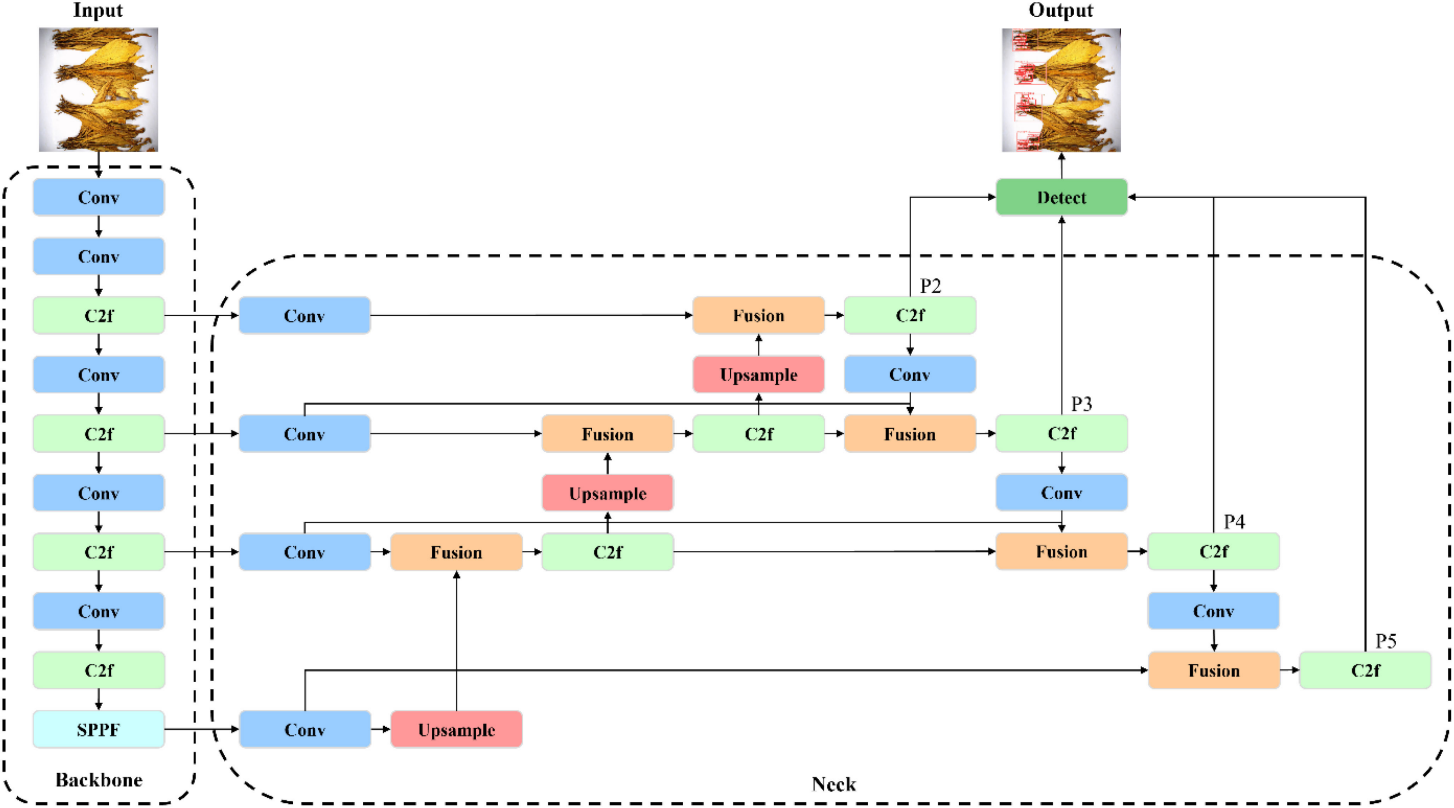
Structure of the YOLO-TobaccoStem model.

To facilitate industrial deployment while ensuring the accuracy of tobacco stem detection, we developed the YOLO-TobaccoStem lightweight model to complete the task of detecting tobacco stems in the tobacco leaf sorting environment. First, a small object detection layer was added to capture more details of small objects and improve the detection accuracy for small objects. Second, given the varying sizes of tobacco stem objects, we enhanced the feature fusion network of the YOLOv8 model to improve its ability to detect multi-scale objects. Lastly, a monotonic focusing mechanism was introduced to the loss function to enhance the model’s accuracy in detecting highly overlapping objects. The overall structure of the YOLO-TobaccoStem model is illustrated in **Figure 6**.

Like the YOLOv8 model, the YOLO-TobaccoStem model also consists of the input end, backbone network, neck network, and detection head. First, tobacco leaf images are resized to a resolution of 640×640 and fed into the input end. The backbone network then extracts and fuses multi-scale features through stacked convolutional (3×3 Conv, Stride 1) and C2f modules, generating hierarchical feature maps. These features are further processed by a Spatial Pyramid Pooling-Fast (SPPF) module to concatenate multi-scale representations, thereby enhancing the model’s multi-scale detection capability. Subsequently, the neck network refines these features using upsampling and fusion operations, while integrating a bidirectional feature pyramid structure to strengthen semantic information integration across scales. The feature map dimensions for P2, P3, P4, and P5 are 160×160, 80×80, 40×40, and 20×20 respectively, with the model utilizing the SiLU activation function. Finally, the detection head predicts bounding box coordinates and class probabilities based on the refined features, outputting detailed target information for detected objects.

#### 2.3.3 Small object detection layer

**Figure 7:**
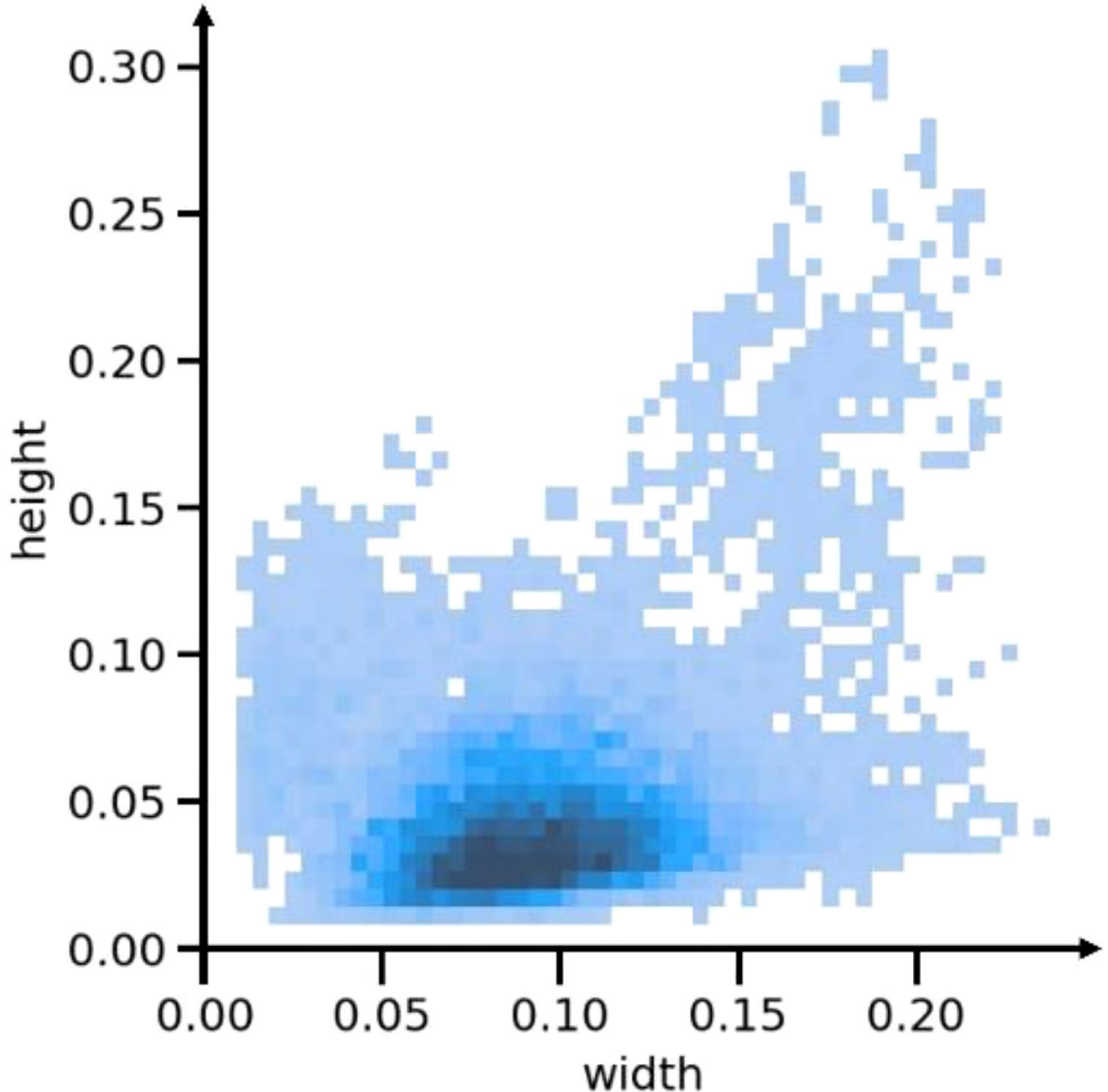
Distribution chart of label sizes.

Due to the presence of overlapping tobacco leaves on the conveyor belt, many tobacco stem objects appear as small objects. Through a detailed analysis of the training set in the tobacco leaf loosening dataset, we plotted the distribution of label sizes, which is illustrated in **Figure 7**. It can be observed that small objects, with sizes ranging between 0.05 and 0.15, constitute a significant proportion.

Since small objects contain fewer pixels in the input image, the information on the feature map is further reduced after downsampling and convolution operations, leading to a loss of details. In YOLOv8, when the input image size is 640×640, the feature map sizes corresponding to the P5, P4, and P3 detection layers are 20×20, 40×40, and 80×80, respectively. This resolution may not be sufficient to retain the details of small tobacco stem objects, resulting in suboptimal detection performance. To address this issue, this study proposes adding a P2 detection layer during the feature extraction and detection stages, with a corresponding feature map size of 160×160. This addition helps to preserve more details of small objects, enabling the model to capture the characteristics of small objects more clearly, thereby improving detection accuracy.

#### 2.3.4 Multi- scale feature fusion

**Figure 8:**
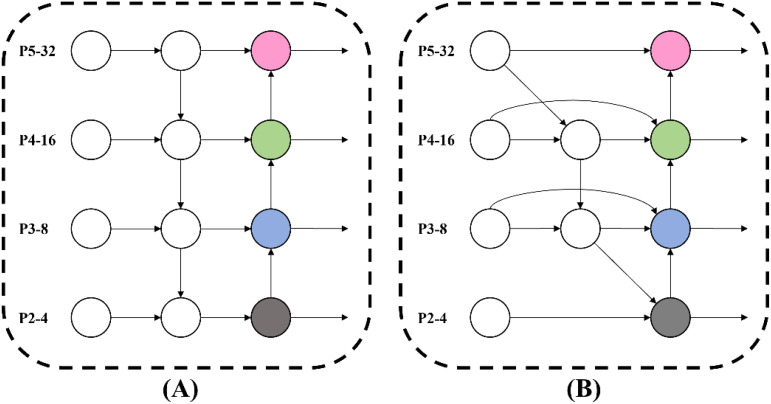
Network structures of FPN-PAN and BiFPN. **(A)** FPN-PAN network structure. **(B)** BiFPN network structure.

During the tobacco leaf sorting process, varying degrees of leaf occlusion result in tobacco stem objects of different sizes within images. Additionally, small objects with limited pixel counts in input images experience further information loss on feature maps after downsampling and convolutional operations, leading to diminished detail retention. These challenges demand enhanced small objects feature extraction and multi-scale information transfer capabilities in detection models. While YOLOv8 primarily utilizes the FPN-PAN structure in its neck network, the differing resolutions of input features often prevent effective integration of multi-scale characteristics. To resolve this, we introduced the BiFPN structure (Tan et al., 2020), where bidirectional connections enable cross-resolution feature propagation. This approach better combines low-level and high-level features, producing feature maps with richer semantic information and thereby improving detection accuracy. The network structures of FPN-PAN and BiFPN are illustrated in **Figure 8**.

BiFPN also adopts an adaptive feature fusion mechanism to better match the needs of different tasks by integrating various input features, thereby improving the effectiveness of feature fusion. The expressions of BiFPN are formulated as Equations 1, 2:


(1) $$\begin{array}{c} P_{x}^{td}=Conv(\frac{w_{1}\cdot P_{x}^{in}+w_{2}\cdot Resize(P_{x+1}^{in})}{w_{1}+w_{2}+Є}) \end{array}$$

(2) $$\begin{array}{c} P_{x}^{out}=Conv(\frac{w_{1}^{\prime }\cdot P_{x}^{in}+w_{2}^{\prime }\cdot P_{x}^{td}+w_{3}^{\prime }\cdot Resize(P_{x-1}^{out})}{w_{1}^{\prime }+w_{2}^{\prime }+w_{3}^{\prime }+Є}) \end{array}$$

where $P_{x}^{td}$ is the intermediate feature at level *x* on the top-down pathway, and $P_{x}^{out}$ is the output feature at level *x* on the bottom-up pathway. All other features are constructed in a similar manner.

By introducing BiFPN to improve the model’s feature pyramid structure, it can not only effectively enhance the model’s ability to integrate multi-scale features but also improve the detection accuracy of tobacco stem objects of different sizes.

#### 2.3.5 Boundary box loss based on monotonic focusing mechanism

The tobacco leaves on the conveyor belt may have overlapping object heights due to multiple tobacco stems being too close, and the features of a single tobacco stem object will be obstructed by other tobacco stem objects. The obstructed tobacco stem objects belong to difficult samples, while the unobstructed tobacco stem objects belong to simple samples. Therefore, during object detection, there may be difficulties in locating bounding boxes, making it difficult to accurately identify difficult samples and resulting in missed detections. The loss function used in YOLOv8 is CIoU Loss (Zheng et al., 2020), and its formula is formulated as Equations 3-7:


(3) $$\begin{array}{c} IoU=\frac{\left|B\;\cap \;B^{gt}\right|}{\left|B\;\cup \;B^{gt}\right|} \end{array}$$

(4) $$\begin{array}{c} v=\frac{4}{\pi^{2}}{\left(arctan\frac{w^{gt}}{h^{gt}}-arctan\frac{w}{h}\right)}^{2} \end{array}$$

(5) $$\begin{array}{c} \alpha =\frac{v}{(1-IoU)+v} \end{array}$$

(6) $$\begin{array}{c} R_{CIoU}=\frac{\rho^{2}(b,b^{gt})}{c^{2}}+\alpha v \end{array}$$

(7) $$\begin{array}{c} L_{CIoU}=1-IoU+\frac{\rho^{2}(b,b^{gt})}{c^{2}}+\alpha v \end{array}$$

where $B^{gt}=(x^{gt},y^{gt},w^{gt},h^{gt})\;$ is the true bounding box, and B=(x,y,w,h) is the predicted bounding box; IoU is the intersection union ratio between predicted bounding box and real bounding box; *v* is used to measure the consistency between the predicted bounding box and the true bounding box aspect ratio; *α* is the weight coefficient; $\rho (b,b^{gt})\;$ is the Euclidean distance between the center point of the predicted bounding box and the true bounding box; *c* is the diagonal length of the smallest enclosing box covering the predicted bounding box and the true bounding box; $R_{CIoU}$ is a penalty term.

However, when faced with highly overlapping tobacco stems in the dataset of scattered tobacco leaves, multiple tobacco stems closely overlap, and their center point distance $\rho (b,b^{gt})$ is very small, which greatly weakens the contribution to the loss function, resulting in limited effectiveness in distinguishing highly overlapping tobacco stems.

To solve the above problem, this study chooses to replace CIoU with WIoU (Tong et al., 2023) as the loss function of the model. WIoU is a loss function based on IoU (Yu et al., 2016), which can reduce the influence of high-quality anchor boxes through a gradient gain allocation strategy, making WIoU more focused on low-quality anchor boxes, thereby improving the detection performance of the model. This study uses the version 2 of WIoU, and its formula is formulated as Equations 8-11:


(8) $$\begin{array}{c} L_{IoU}=1-IoU \end{array}$$

(9) $$\begin{array}{c} R_{WIoU}=exp(\frac{{\left(x-x_{gt}\right)}^{2}+{\left(y-y_{gt}\right)}^{2}}{{\left(W_{g}^{2}+H_{g}^{2}\right)}^{*}}) \end{array}$$

(10) $$\begin{array}{c} L_{WIoUv1}=R_{WIoU}L_{IoU} \end{array}$$

(11) $$\begin{array}{c} L_{WIoUv2}={\left(\frac{L_{IoU}^{*}}{\bar{L_{IoU}}}\right)}^{\gamma }L_{WIoUv1} \end{array}$$

where $R_{WIoU}$ is a penalty term; $W_{ɡ}$ , $H_{ɡ}$ are the size of the smallest enclosing box; ${\left(\frac{L_{IoU}^{*}}{\bar{L_{IoU}}}\right)}^{\gamma }$ is the monotonic focusing coefficient.

WIoU v2 has designed a monotonic focusing mechanism for cross entropy, which effectively reduces the contribution of simple samples to the loss value. This allows the model to focus on difficult samples, thereby achieving better detection results for occluded tobacco stem objects.

### 2.4 Tobacco leaf loosening rate detection algorithm

By utilizing the trained YOLO-TobaccoStem model, the loosened tobacco leaves in the sorting scenario are detected and identified, and the model will output an array as shown in Equation 12:


(12) $$\begin{array}{c} N\times [x_{1},y_{1},x_{2},y_{2},conf,class] \end{array}$$

where *N* is the number of predicted bounding boxes in the image; $x_{1},y_{1}$ is the coordinates of the top-left corner of the predicted bounding box; $x_{2},y_{2}$ is the coordinates of the bottom-right corner of the predicted bounding box; conf is the confidence score of the predicted object in that classification; and class is the class information of the predicted object.

Accordingly, this study designs a tobacco leaf loosening rate detection algorithm. Firstly, the coordinates $x_{1},y_{1},x_{2},y_{2}$ are used to determine the positions of all predicted bounding boxes in the image, and the class is used to determine whether the object is a single tobacco stem or overlapping tobacco stems. Then, the relative positions of the bounding boxes for the two types of objects are analyzed. All single tobacco stem objects overlapping with the same overlapping tobacco stems object are considered to be part of the same bunch of tobacco leaves, and their quantity is counted. This count is then used to determine the number of stems in a bunch, denoted as *x*. Finally, the tobacco leaf loosening rate is calculated using a predefined parameter λ, as shown in Equation 13:


(13) $$\begin{array}{c} R_{sanba}=\frac{\lambda }{x}\times 100\% \end{array}$$

**Figure 9:**
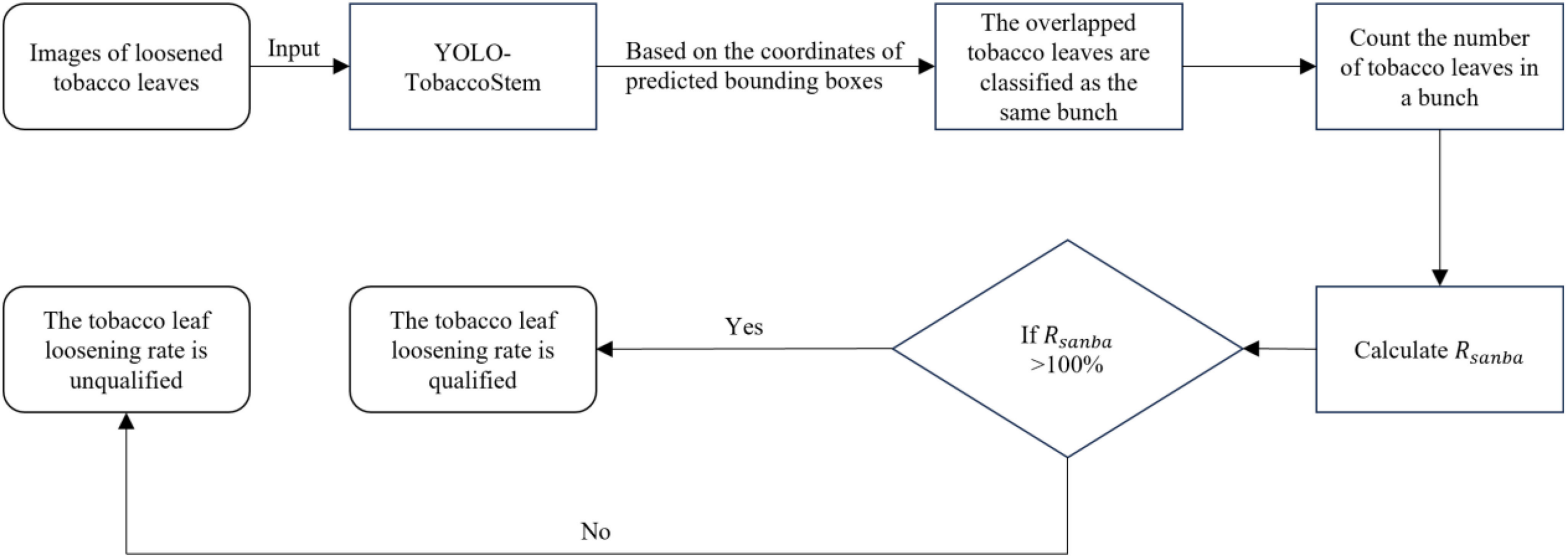
Tobacco leaf loosening rate detection algorithm flowchart.

The predefined parameter *λ* represents the maximum number of stems that a normal bunch of tobacco leaves can contain. This parameter can be set in advance by field staff at the tobacco leaf sorting site. If *x* is lower than *λ*, it is considered a normal situation; if *x* is higher than *λ*, it is considered an abnormal situation. When the tobacco leaf loosening rate $R_{sanba}>$ 100%, it indicates a good loosening effect, meaning a fully loosened state that meets the actual production needs. Conversely, if $R_{sanba}$ ≦100%, it indicates a poor loosening effect, and the workers’ operations need timely guidance. **Figure 9** shows the flow chart of the tobacco leaf loosening rate detection algorithm.

## 3 Experiments and results

### 3.1 Training environment

The model training was conducted on a Windows 11 operating system. The experimental computational resources included an NVIDIA GeForce RTX 4090 GPU with 24 GB of memory, an Intel(R) Core(TM) i7-13700K CPU at 3.40 GHz, and 32 GB of RAM. Model construction, training, and evaluation were all carried out in the Python programming environment using the PyCharm integrated development environment, with PyTorch 2.0.0 as the deep learning framework and CUDA 11.7 for parallel computing.

Based on the study by Srinivasu et al. (2025) and empirical testing results on our dataset, the hyperparameter settings utilized during model training are systematically presented in **Table 2**. To ensure fairness in training, no pre-trained weights were loaded during the model training process.

**Table 2:** Hyperparameter settings.

Hyperparameter	Value
Optimizer	SGD
Momentum	0.937
Weight decay	0.0005
Initial learning rate	0.01
Final learning rate	0.01
Epochs	500
Batch size	16
Workers	8

### 3.2 Evaluation metrics

Since the tobacco leaf loosening rate detection algorithm proposed in Section 2.4 heavily relies on the accuracy of the object detection model, it is essential to evaluate the performance of the YOLO-TobaccoStem model. This study employs Precision (P), Recall (R), and mean Average Precision (mAP) as the evaluation metrics for the model’s detection performance. The following definitions are used:

TP (True Positives): the number of correctly predicted positive samples; FP (False Positives): the number of incorrectly predicted positive samples; FN (False Negatives): the number of actual positive samples incorrectly predicted as negative.

Precision is the proportion of correctly predicted positive samples among all samples predicted as positive by the model. The calculation formula is shown in Equation 14:


(14) $$\begin{array}{c} Precision=\frac{TP}{TP+FP} \end{array}$$

Recall is the proportion of correctly predicted positive samples among all actual positive samples. The calculation formula is shown in Equation 15:


(15) $$\begin{array}{c} Recall=\frac{TP}{TP+FN} \end{array}$$

F1-Score is the harmonic mean of precision and recall, and it’s an indicator used to measure the accuracy of binary classification. Its maximum value is 1, and its minimum value is 0. The larger the value, the better the classification effect. The calculation formula is shown in Equation 16:


(16) $$\begin{array}{c} F_{1}=2\times \frac{Precision\times Recall}{Precision+Recall} \end{array}$$

AP (Average Precision) is the area under the curve calculated by plotting the P-R curve (Precision-Recall curve), while mAP is the average value of AP for all categories. The calculation formula is shown in Equation 17:


(17) $$\begin{array}{c} mAP=\frac{1}{N}\sum_{i=1}^{N}AP_{i} \end{array}$$

where *N* is the number of categories. In this paper, *N*=2, and $AP_{i}$ is the AP of the *i*-th category.

In addition to the above metrics for evaluating model detection performance, the model can also be assessed using FPS (Frames Per Second), the parameters, and the size of the weight file. FPS indicates the number of image frames the model can process per second, which reflects the model’s processing speed. A higher FPS indicates better real-time performance. This makes the model more suitable for tasks requiring real-time detection. The parameters and weight file size reflect the model’s complexity and hardware resource requirements. More parameters and weight file size indicate a more complex model and higher hardware requirements for deployment.

Using these evaluation metrics, we can comprehensively assess the model’s performance and select the object detection model that best contributes to this study.

### 3.3 Ablation experiment

This study conducted ablation experiments on the test set of the tobacco leaf loosening dataset to verify the contribution of each module to the overall performance of the YOLO-TobaccoStem model. The results of the ablation experiments are illustrated in **Table 3**. We used YOLOv8n as the baseline model, which has a mAP0.5 of 93.4%, a mAP0.5-0.95 of 66.1%, parameters of 3.01 M, and a weight file size of 6.3 MB. Various module combination experiments were conducted without using pretrained weights to determine their individual effects on the model.

**Table 3:** Ablation experiment results.

Group	A	B	C	D	E	F	G	H
P2		✓			✓	✓		✓
BiFPN			✓		✓		✓	✓
WIoU v2				✓		✓	✓	✓
P(%)	93.7	92.5	93.9	93.4	91.8	93.6	93.8	92.6
R(%)	88.6	89.1	90.4	90.5	89.5	90.0	90.2	91.6
mAP0.5(%)	93.4	94.3	94.0	94.4	94.9	94.5	94.5	95.2
mAP0.5-0.95(%)	66.1	67.5	67.0	68.4	68.4	67.2	66.9	68.3
FPS	213	145	179	213	167	145	179	167
Parameters(M)	3.01	2.92	1.99	3.01	2.19	2.92	1.99	2.19
Weight File Size(MB)	6.3	6.3	4.3	6.3	4.8	6.3	4.3	4.8

First, Group B experimented with the addition of the P2 small object detection layer, showing a 0.9% improvement in mAP0.5 and a 1.4% improvement in mAP0.5-0.95, with no significant change in the parameters and weight file size. Groups E and F added the P2 small object detection layer on top of other module improvements, demonstrating that the P2 detection layer effectively retains more details of small objects and thus improves detection accuracy. However, combining it with BiFPN increased the parameters by 0.2 M and the weight file size by 0.5 MB. This indicates that adding the P2 small object detection layer can enhance detection accuracy without significantly increasing model complexity.

Next, we tested BiFPN in Group C, which improved the model by 0.6% in mAP0.5 and 0.9% in mAP0.5-0.95, while reducing the parameters by 1.02 M and the weight file size by 2 MB. Combining BiFPN with other modules also reduced the parameters and weight file size without significantly compromising detection accuracy. This result indicates that BiFPN can effectively achieve a lightweight improvement while enhancing detection accuracy.

Then, experiments with WIoU v2 in Groups D, F, and G showed that it improved mAP0.5 by 1%, 0.2%, and 0.5%, respectively. Since WIoU v2 is a loss function replacement, the parameters and weight file size remained unchanged. Therefore, WIoU v2 is a crucial improvement that enhances detection accuracy without increasing model complexity.

Finally, the complete YOLO-TobaccoStem model in Group H showed a 1.8% improvement in mAP0.5 and a 2.2% improvement in mAP0.5-0.95, compared to the baseline YOLOv8n model, with a 27.2% reduction in parameters and a 23.8% reduction in weight file size. Although the improved model showed a 1.1% decline in precision compared to the baseline, it achieved a 3.0% improvement in recall rate. This trade-off demonstrates that the marginal precision loss is operationally acceptable. While the model’s FPS experienced a measurable reduction, its maintained FPS of 167 frames/s remains fully capable of meeting real-time detection requirements. This demonstrates that the proposed model improvements are both effective and efficient.

**Figure 10:**
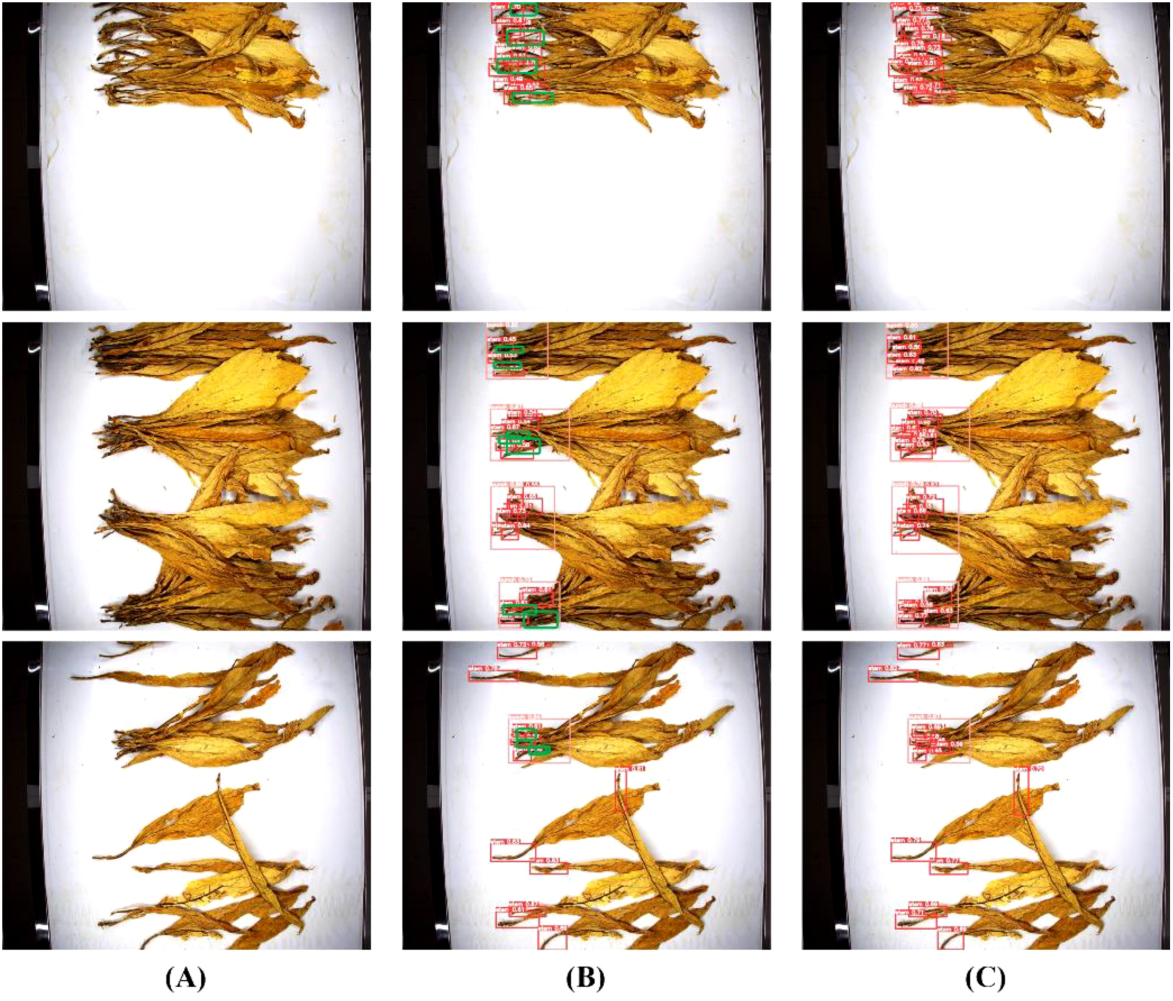
Detection performance comparison between YOLO-TobaccoStem and YOLOv8n. **(A)** Original image. **(B)** YOLOv8n detection result. **(C)** YOLO-TobaccoStem detection result.

The detection performance comparison between YOLO-TobaccoStem and YOLOv8n is illustrated in **Figure 10**. The green boxes represent tobacco stem objects detected by YOLO-TobaccoStem but not by YOLOv8n. It is evident that YOLO-TobaccoStem outperforms the baseline model YOLOv8n.

**Figure 11:**
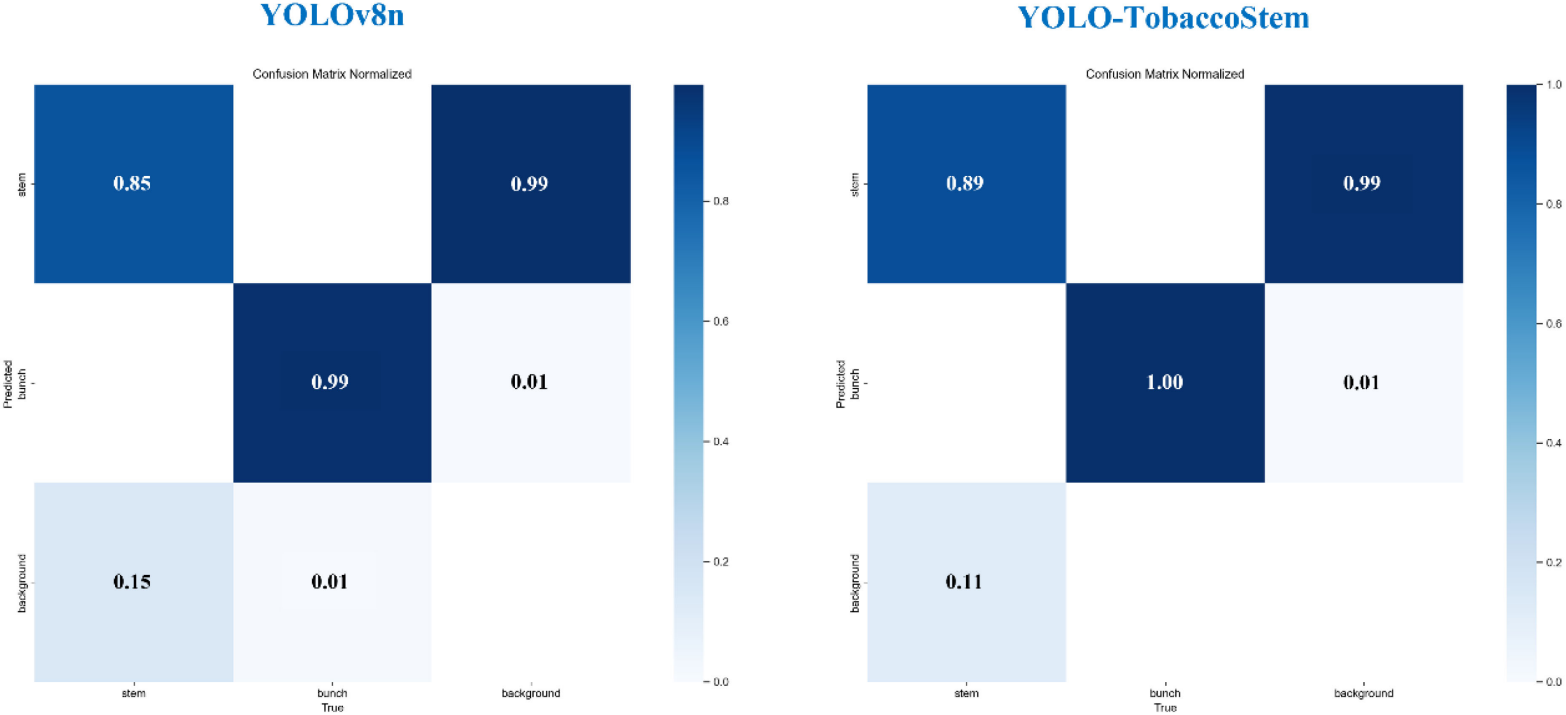
Confusion matrices of YOLOv8n and YOLO-TobaccoStem.

**Figure 11** illustrates the confusion matrices of YOLOv8n and YOLO-TobaccoStem trained on the tobacco leaf loosening dataset. In the confusion matrices, the x-axis denotes the ground truth classes (the actual annotated categories in the dataset), while the y-axis represents the predicted classes (the model’s classification outputs). The results indicate that YOLO-TobaccoStem also demonstrates better performance in terms of detection accuracy.

**Figure 12:**
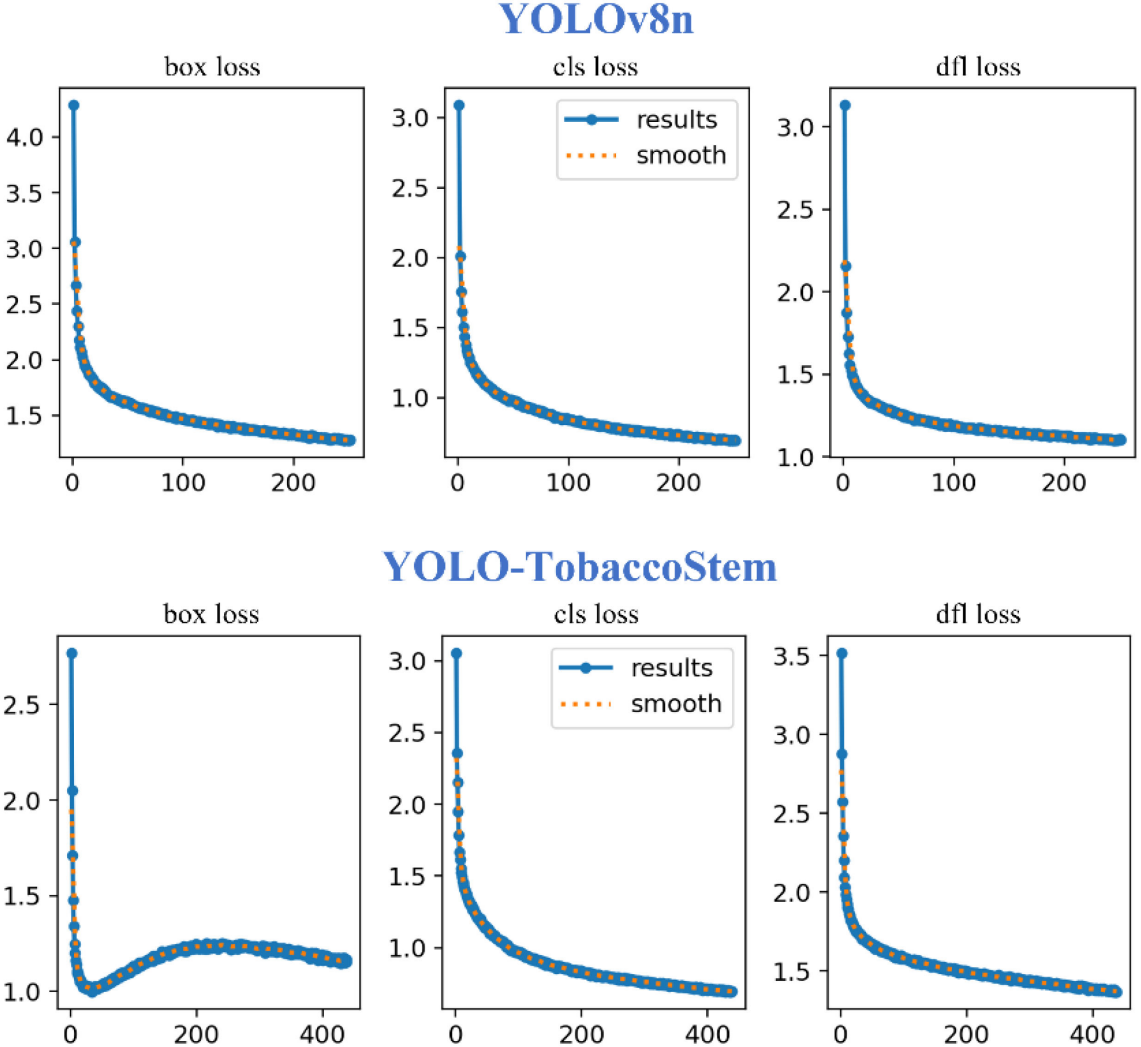
Loss curves of YOLOv8n and YOLO-TobaccoStem.

**Figure 12** illustrates the loss curves of YOLOv8n and YOLO-TobaccoStem. The curves demonstrate comparable cls loss between both models. Regarding dfl loss, YOLOv8n shows a marginally higher value than YOLO-TobaccoStem, though the difference remains minor. Notably, YOLO-TobaccoStem achieves a significantly lower box loss after convergence, thereby attaining a higher detection accuracy. Thus, YOLO-TobaccoStem demonstrates superior detection performance compared to the baseline YOLOv8n model.

In summary, the ablation experiments demonstrate the significant improvements brought by the P2 small object detection layer, BiFPN, and WIoU v2 in the proposed model. These enhancements not only improve detection accuracy but also make the model more lightweight. Furthermore, the YOLO-TobaccoStem model achieves a detection accuracy of 95.2% while using minimal hardware resources, making it suitable for real deployment in tobacco leaf sorting scenarios.

### 3.4 Comparative experiment

In order to test the performance of the YOLO-TobaccoStem model proposed in this paper, comparative experiments were conducted with all mainstream YOLO models and RT-DETR models currently available. **Table 4** illustrates a comparison of the experimental results between different models.

**Table 4:** Comparative experiments results of different models.

Model	P (%)	R (%)	mAP0.5 (%)	F1-Score	FPS	Parameters (M)	Weighted file size (MB)
YOLOv3	94.2	91.6	94.2	92.9	34	61.53	123.5
YOLOv3-tiny	90.9	85.6	90.0	88.2	84	8.67	17.4
YOLOv3-spp	94.4	91.2	94.4	92.8	39	62.58	156.1
YOLOv5n	91.5	87.5	92.6	89.5	90	1.76	4.1
YOLOv5s	92.3	89.0	93.4	90.6	47	7.02	14.4
YOLOv5m	94.2	89.8	93.4	91.9	35	20.86	42.2
YOLOv5l	94.8	91.1	94.2	92.9	36	46.11	92.8
YOLOv5x	94.6	90.7	94.1	92.6	35	86.18	173.1
YOLOv6n	93.3	89.0	93.1	91.1	182	4.23	8.7
YOLOv6s	94.3	90.9	94.3	92.6	96	16.30	32.9
YOLOv6m	94.3	91.7	94.5	93.0	62	51.98	104.4
YOLOv6l	94.9	91.2	94.2	93.0	38	110.86	222.2
YOLOv6x	94.5	94.6	94.3	94.5	24	172.98	346.5
YOLOv7	94.6	91.0	94.0	92.8	33	36.49	74.8
YOLOv7-tiny	92.3	90.2	93.5	91.2	68	6.01	12.3
YOLOv8n	93.7	88.6	93.4	91.1	213	3.01	6.3
YOLOv8s	93.7	91.9	94.5	92.8	137	11.13	22.5
YOLOv8m	94.2	92.2	94.8	93.2	91	25.84	52.0
YOLOv8l	94.2	92.9	94.7	93.5	66	43.61	87.7
YOLOv8x	94.4	93.0	94.7	93.7	38	68.13	136.7
YOLOv9	93.9	92.5	95.2	93.2	41	60.76	122.3
YOLOv9-c	94.8	91.6	94.6	93.2	44	50.96	102.8
Yolov10n	88.8	88.2	92.1	88.5	167	2.27	5.8
Yolov10s	90.0	88.6	92.5	89.3	159	7.22	16.5
Yolov10m	90.3	89.7	93.3	90.0	104	15.31	33.5
Yolov10b	89.3	90.3	93.1	89.8	87	19.01	41.5
Yolov10l	90.1	89.9	93.3	90.0	69	24.31	52.2
Yolov10x	91.4	88.3	93.1	89.8	57	29.40	64.1
Yolov11n	89.3	87.5	91.4	88.4	185	2.58	5.5
Yolov11s	90.2	90.3	92.6	90.2	152	9.41	19.2
Yolov11m	91.8	88.8	92.5	90.3	128	20.03	40.5
Yolov11l	89.7	89.2	92.6	89.4	89	25.28	51.2
Yolov11x	91.7	89.6	92.7	90.6	65	56.83	114.4
Yolov12n	90.4	87.3	91.4	88.8	145	2.51	5.5
Yolov12s	89.6	88.9	92.6	89.2	115	9.07	18.7
Yolov12m	90.2	89.0	92.6	89.6	110	19.58	39.8
Yolov12l	91.0	89.3	92.3	90.1	72	25.76	52.5
RT-DETR (ResNet-18)	87.3	89.5	93.3	88.4	123	19.87	40.5
RT-DETR (ResNet-34)	87.7	80.5	92.3	83.9	98	31.11	63.0
RT-DETR (ResNet-50)	87.2	91.6	93.4	64.4	80	41.96	86.1
Ours	92.6	91.6	95.2	92.1	167	2.19	4.8

**Table 4** clearly shows that the YOLO TobaccoStem model proposed in this paper exhibits excellent performance in mAP0.5, parameters, weight file size, and FPS.

In terms of mAP0.5, our model achieved the best result of 95.2% among all models, with the highest detection accuracy among all mainstream YOLO models. YOLOv9 (Wang et al., 2024), which has the same detection accuracy as our model but has 27.4 times more parameters and a weight file size that is also 25.5 times larger than our model’s. Therefore, the comprehensive performance of our model is much better than YOLOv9. Although our model did not achieve the best results in terms of parameters and weight file size, it is only second to YOLOv5n and the difference is not significant. Moreover, the detection accuracy of our model is 2.6% higher than YOLOv5n, and the overall performance is better. In terms of FPS, our model is second only to the baseline model YOLOv8n, with an FPS as high as 167 frames/s, which is fast enough to cope with most tasks that require real-time detection. Although YOLO-TobaccoStem does not achieve leading performance in precision, recall, or F1-Score, it still outperforms the majority of comparative models. Moreover, when compared to models with superior performance in these three metrics, our model demonstrates greater advantages in both mAP and model size, while exhibiting stronger suitability for accomplishing the target task defined in this study.

**Figure 13:**
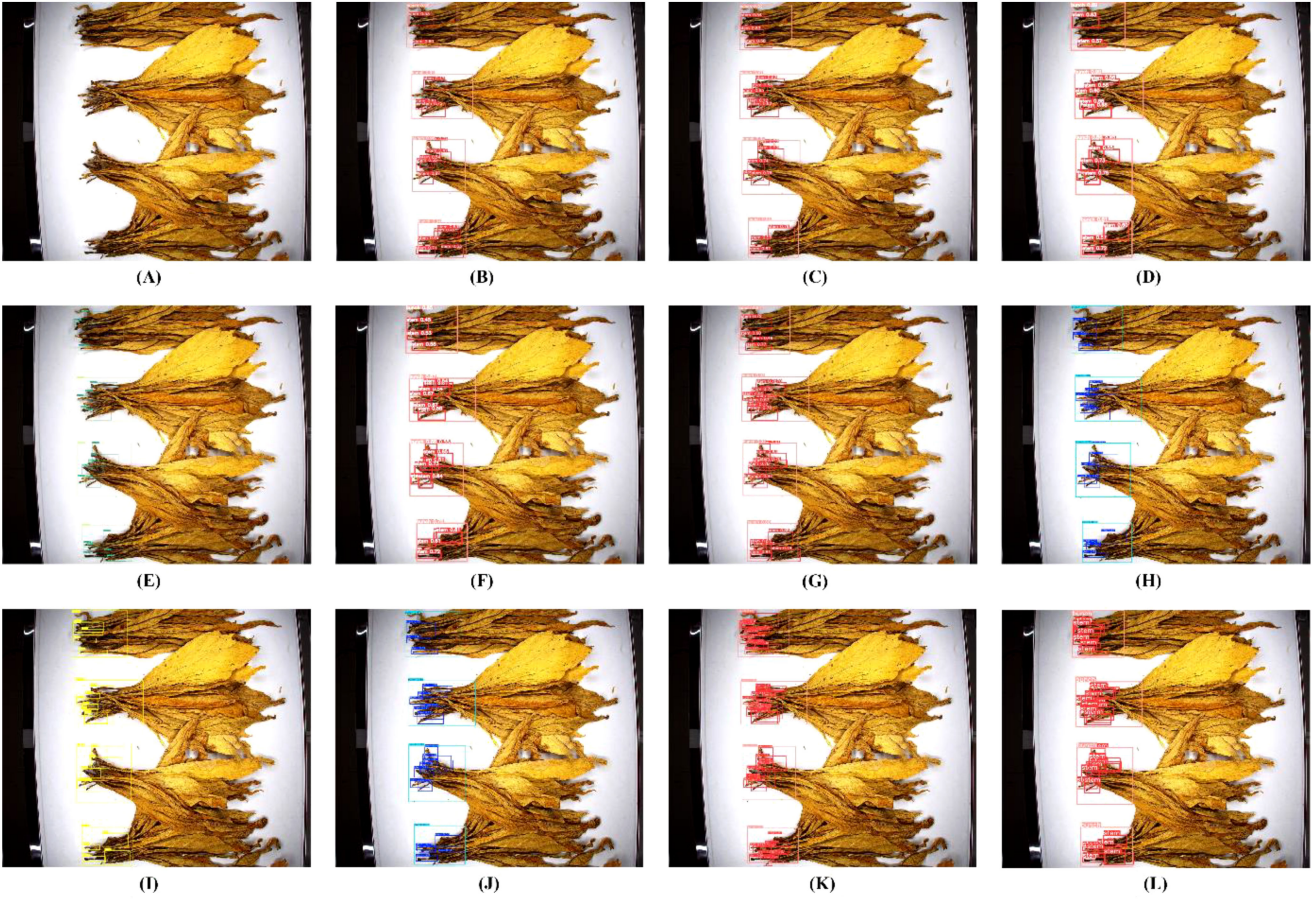
Comparison of detection performance of different models. **(A)** For the original image. **(B)** For YOLOv3-tiny detection result. **(C)** For YOLOv5n detection result. **(D)** For YOLOv6n detection result. **(E)** For YOLOv7-tiny detection result. **(F)** For YOLOv8n detection result. **(G)** For YOLOv9-c detection result. **(H)** For YOLOv10n detection result. **(I)** For YOLOv11n detection result. **(J)** For YOLOv12n detection result. **(K)** For RT-DETR(ResNet-34) detection result. **(L)** For YOLO-TobaccoStem detection result.

**Figure 13** visually displays the detection results among different models. It can be clearly seen that when detecting bunches of tobacco leaves, all models can detect overlapping tobacco stem objects. However, for the detection of a single tobacco stem, YOLOv3 tiny, YOLOv5n, and YOLOv6n all have a large number of missed detections, while YOLOv7 tiny, YOLOv8n, YOLOv9-c, YOLOv10n, YOLOv11n, YOLOv12n and RT-DETR(ResNet-18) perform relatively well. Nonetheless, compared with the detection performance of the YOLO-TobaccoStem model proposed in this study, there is still a certain degree of missed detections.

Overall, through comparative experiments with mainstream YOLO models and RT-DETR models, significant improvements in performance based on YOLOv8n have been achieved in this study. The proposed YOLO-TobaccoStem model demonstrates higher detection accuracy and a smaller model size compared to other models, making it more suitable for real-time detection tasks without encountering resource constraints during deployment. These findings highlight the substantial advantages and potential of the proposed model in the field of tobacco stem loosening rate detection.

## 4 Discussion

### 4.1 The impact of different loss functions on the model

**Figure 14:**
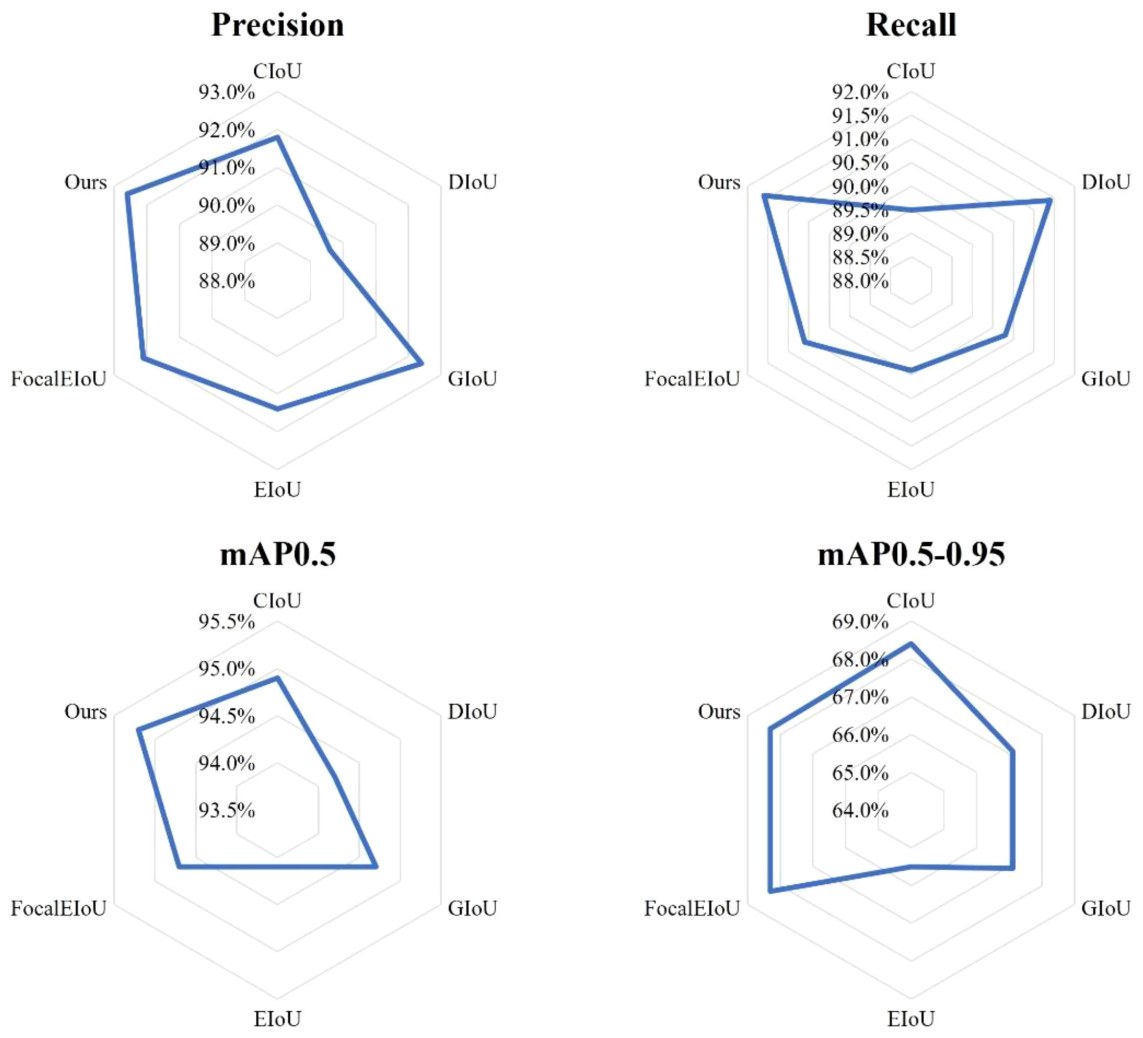
Detection performance of different loss functions.

In the proposed YOLO-TobaccoStem model, WIoU v2 is used to address the issue of poor detection performance on overlapping tobacco stem objects. To demonstrate that WIoU v2 is the most suitable loss function, comparative experiments were conducted with CIoU, DIoU, GIoU (Rezatofighi et al., 2019), EIoU (Zhang et al., 2022), and FocalEIoU. The experimental results are illustrated in **Figure 14**.

GIoU aims to solve the problem of zero gradient when there is no overlap between two objects. It considers not only the overlapping region of the object boxes but also the non-overlapping regions, providing a better measure of overlap. DIoU addresses the issue of undetectable relative positions when object boxes have an inclusion relationship. By directly minimizing the distance between two object boxes, it achieves faster convergence. CIoU is the loss function currently used in mainstream YOLO models. It incorporates the aspect ratio of the object boxes to solve the problem of loss calculation when the center points of the object boxes are the same in DIoU. EIoU and FocalEIoU decompose the aspect ratio loss terms into differences between the predicted width and height and the minimum enclosing box width and height, respectively. This accelerates convergence and improves regression accuracy. FocalEIoU also introduces Focal Loss to address the issue of sample imbalance in the bounding box regression task, focusing more on high-quality anchor boxes. However, for the tobacco leaf loosening dataset used in this study, the focus of the above loss functions is not as suitable as WIoU v2.

From **Figure 14**, it is evident that compared to other loss functions, WIoU v2 used in the YOLO-TobaccoStem model achieves the best results in Precision, Recall, and mAP0.5, with values of 92.6%, 91.6%, and 95.2%, respectively. Although its performance in mAP0.5-0.95 is not the highest, it is second only to CIoU. Based on these results, it can be concluded that WIoU v2 has an advantage over other loss functions in detecting highly overlapping tobacco stem objects.

### 4.2 Analysis of the effectiveness of the tobacco leaf loosening rate detection algorithm

In this study, the trained YOLO-TobaccoStem model was used to detect tobacco leaf loosening images in the test set. The output results of the YOLO-TobaccoStem model were then fed into the tobacco leaf loosening rate detection algorithm (detailed in Equation 13; **Figure 9**) to obtain the tobacco leaf loosening rate detection results. The parameter *λ* of the algorithm was set to 8, meaning that a bunch of tobacco leaves containing fewer than 8 leaves was considered as qualified, while a bunch containing 8 or more leaves was considered unqualified. Using this method, 37 bunches of tobacco leaves were classified into 25 positive samples and 12 negative samples, where positive samples were unqualified bunches of tobacco leaves, and negative samples were qualified bunches of tobacco leaves.

Since the confidence score of the YOLO-TobaccoStem model affects the results of the tobacco leaf loosening rate detection algorithm, the confidence score of the YOLO-TobaccoStem model was continuously adjusted and tested. The experimental data obtained are illustrated in **Table 5**.

**Table 5:** Results of tobacco leaf loosening rate detection algorithm under different confidence scores.

Confidence	TP	TN	FP	FN
0.25	24	6	9	2
0.30	23	8	6	3
0.35	17	10	3	9
0.40	9	12	1	17
0.45	5	12	0	21

**Figure 15:**
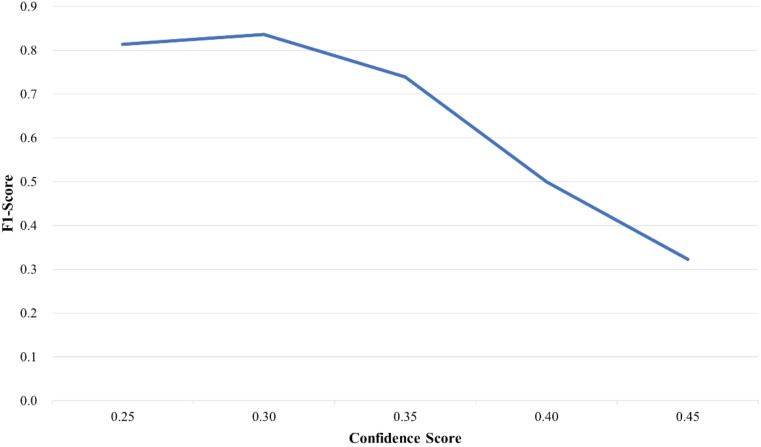
F1-Score of tobacco leaf loosening rate detection algorithm under different confidence scores.

Based on the experimental data in **Table 5**, the F1-Score of the tobacco leaf loosening rate detection algorithm at different confidence scores can be derived, as illustrated in **Figure 15**. When the confidence score is 0.30, the tobacco leaf loosening rate detection algorithm achieves the highest F1-Score of 0.836. Therefore, 0.30 is determined to be the most suitable confidence score.

**Figure 16:**
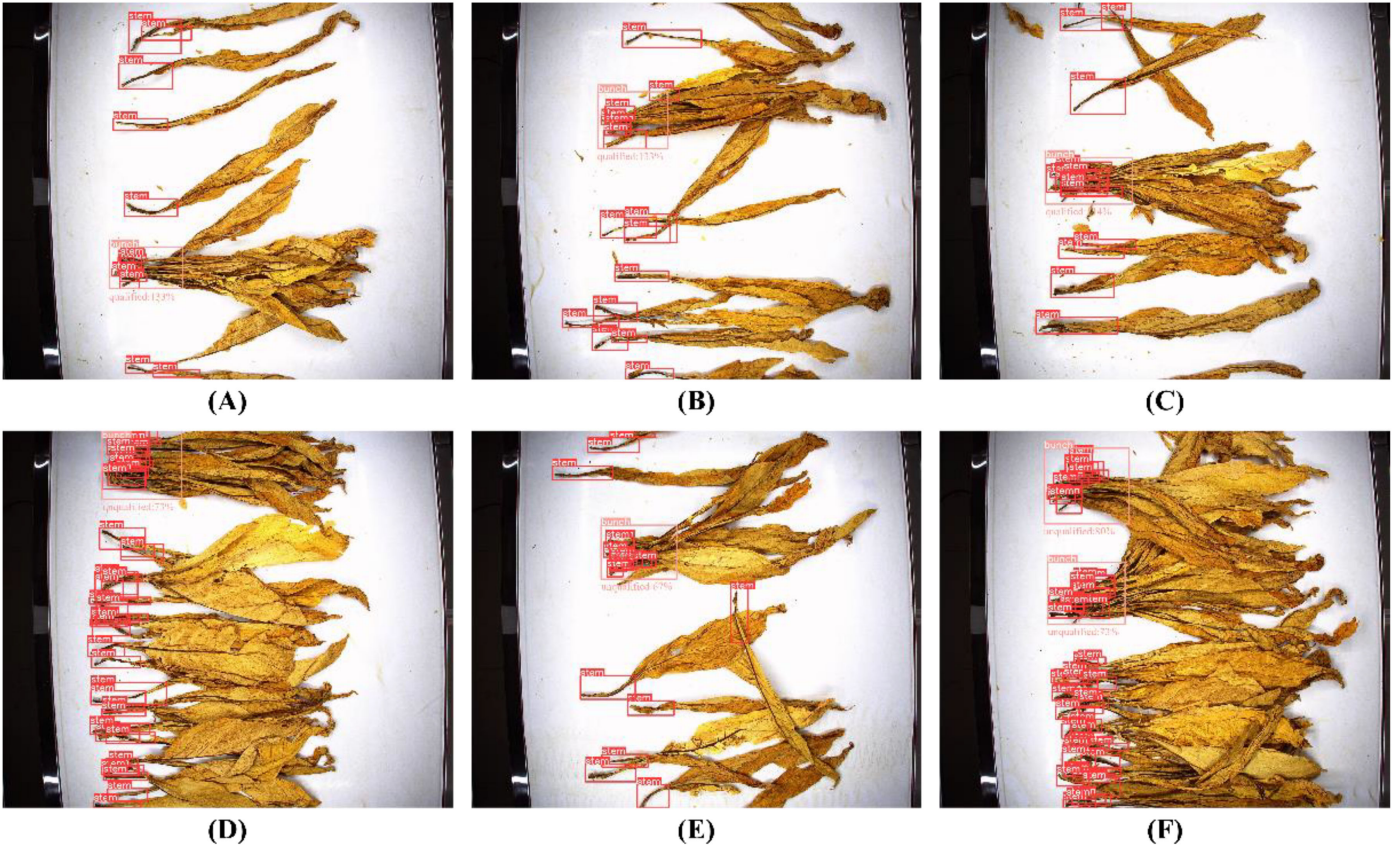
Detection result images of tobacco leaf loosening rate detection algorithm. **(A–C)** Tobacco leaves with qualified loosening. **(D–F)** Tobacco leaveswith unqualified loosening.

Thus, with the model confidence score set at 0.30, the actual detection results of the tobacco leaf loosening rate detection algorithm are shown in **Figure 16**. In the figures, (A)(B)(C) show tobacco leaves with qualified loosening, indicated as “Qualified,” while (D)(E)(F) show tobacco leaves with unqualified loosening, indicated as “Unqualified”.

The detection results of the tobacco leaf loosening rate detection algorithm and the actual conditions of the test set are compared, as illustrated in **Table 6**. In this table, “Actual” indicates the true loosening condition of the bunched tobacco leaves in the test set, where “True” represents qualified loosening and “False” represents unqualified loosening. “Detection result” shows the results from the tobacco leaf loosening rate detection algorithm, and “Correct or not” indicates whether the detection result is correct, with “Correct” indicating correct detection and “Incorrect” indicating incorrect detection.

**Table 6:** Results of tobacco leaf loosening rate detection algorithm.

Bunch number	Actual	Detection result	Correct or not	Bunch number	Actual	Detection result	Correct or not
1	False	False	Correct	20	False	False	Correct
2	False	False	Correct	21	False	False	Correct
3	False	False	Correct	22	False	False	Correct
4	False	False	Correct	23	False	False	Correct
5	True	False	Incorrect	24	False	False	Correct
6	False	False	Correct	25	True	True	Correct
7	True	False	Incorrect	26	False	False	Correct
8	False	False	Correct	27	False	False	Correct
9	False	False	Correct	28	True	True	Correct
10	False	False	Correct	29	True	True	Correct
11	False	True	Incorrect	30	True	True	Correct
12	True	True	Correct	31	True	True	Correct
13	True	True	Correct	32	False	False	Correct
14	False	False	Correct	33	False	False	Correct
15	False	False	Correct	34	False	False	Correct
16	False	False	Correct	35	True	False	Incorrect
17	False	False	Correct	36	True	True	Correct
18	True	False	Incorrect	37	False	True	Incorrect
19	False	False	Correct	Wrong bunch detection			3

From **Table 6**, the tobacco leaf loosening rate detection algorithm performs well. A total of 40 bunches of tobacco leaves were detected, with 31 bunches correctly detected and 9 bunches incorrectly detected, achieving an overall detection accuracy of 77.5%. The detection accuracy for unqualified and qualified bunches of tobacco leaves was 79.3% and 72.7%, respectively, indicating a high level of performance. Through the experiments and manual verification, it is proven that the tobacco leaf loosening rate detection algorithm proposed in this study has practical application value and holds significant potential for research in related fields.

## 5 Conclusion

In the tobacco leaf sorting process, the tobacco leaf loosening stage is crucial. The quality of the tobacco leaf loosening directly affects the ability to successfully sort different grades of tobacco leaves and non-tobacco related materials. Since this stage heavily relies on the subjective judgment of workers, it is important to propose a method for detecting the rate of tobacco leaf loosening. Real-time monitoring of the rate of tobacco leaf loosening can significantly reduce the occurrence of substandard loosening. With the rapid advancement of object detection technology, this paper proposes a method for detecting the rate of tobacco leaf loosening by combining it with advanced object detection algorithms. The research confronted four challenges: first, the inability to obtain standardized image data under industrial tobacco leaf sorting scenarios; second, the absence of dedicated object detection models for identifying dispersed tobacco leaf bundles; third, the absence of a dataset for tobacco leaf loosening; fourth, the lack of algorithms for quantitatively evaluating tobacco leaf loosening rates.

To tackle these challenges, this study involved extensive efforts and innovations. First, we constructed a darkroom image acquisition system, which enabled us to collect standardized images in the tobacco leaf sorting environment. Second, we built a tobacco leaf loosening dataset, which consists of 2955 images, including 48109 single tobacco stem objects and 907 overlapping tobacco stem objects. This effort filled the gap in the insufficient availability of datasets in the field of tobacco leaf loosening research. Next, we made targeted improvements to the YOLOv8 object detection model. By optimizing the small object detection layer, feature fusion network, and loss function, we developed the YOLO-TobaccoStem model specifically for tobacco stem detection. On our dataset, YOLO-TobaccoStem achieved a mAP of 95.2%, outperforming all other mainstream YOLO models and RT-DETR models while maintaining minimal parameters and model size, ensuring that the model can be deployed in real-world scenarios without hardware resource issues. Finally, using the detection results from the YOLO-TobaccoStem model, we constructed a tobacco leaf loosening rate detection algorithm. This algorithm analyzes the relative positions of objects to determine if the tobacco leaf loosening rate is satisfactory. In experiments conducted on the test set, the algorithm achieved an F1-Score of 0.836, demonstrating its potential for practical deployment.

In summary, the YOLO-TobaccoStem model achieves the highest detection accuracy among mainstream YOLO variants and RT-DETR models while maintaining a compact model size, thereby meeting practical deployment requirements. Experimental validation of the tobacco leaf loosening rate detection algorithm further confirms the practical applicability of our methodology. Additionally, the curated and publicly released dataset serves as a foundational data resource to support related research in tobacco-related research fields. However, there are still some issues and areas for improvement in this method. For example, further optimization of the tobacco leaf loosening rate detection algorithm could enhance its precision and recall. Future research will focus on upgrading and improving the tobacco leaf loosening rate detection algorithm, as well as enhancing the accuracy of the object detection model through additional image preprocessing techniques, thus providing a better data foundation for the tobacco leaf loosening rate detection algorithm.

## Data Availability

The datasets presented in this study can be found in online repositories. The names of the repository/repositories and accession number(s) can be found below: https://github.com/UtahaForever/tobacco/tree/f446ae2da165da48524a7c862ff2bd0f9e823061.
